# Abundance, Functional, and Evolutionary Analysis of Oxalyl-Coenzyme A Decarboxylase in Human Microbiota

**DOI:** 10.3389/fmicb.2020.00672

**Published:** 2020-04-23

**Authors:** Tao Jiang, Wenwei Chen, Linsheng Cao, Yanfeng He, Huiliang Zhou, Houping Mao

**Affiliations:** Department of Urology, The First Affiliated Hospital of Fujian Medical University, Fuzhou, China

**Keywords:** oxalyl-coenzyme A decarboxylase, evolution, microbiota, OXCs, oxalic acid, oxalate salts

## Abstract

Oxalic acid and its oxalate salts have been linked to kidney stones and other health problems and about 80% kidney stones are made up of calcium oxalate. Oxalyl coenzyme A decarboxylase (OXC) is a key enzyme in the catabolism of oxalate. In this study, we performed bioinformatic and biochemical analysis of OXC. First, we mined the OXC sequences from a public protein database and collected 1396 putative OXC sequences. These sequences were widely spread and mainly distributed in Actinobacteria, Alphaproteobacteria, Gammaproteobacteria, and Betaproteobacteria and classified into seven clusters. The phylogenetic relationship and evolutionary rate of the 7 clusters showed that OXC are highly conserved. Second, the abundance of the different clusters of OXC was explored in 380 human microbiome datasets, which showed that OXCs in Cluster 1 were relatively high in the gut while OXCs in Clusters 2–4 were relatively enriched in the vagina. Third, we measured the activity of one OXC from *Mycobacterium mageritense* (OXCmm) in Cluster 3, in which there was no experimentally characterized enzymes. Mutation analysis showed that OXCmm shared the same active sites with the OXC from *Oxalobacter formigenes*. Taken together, this analysis provides a better insight into the distribution and catalysis of OXC and further potential alternative application of OXC active bacteria as probiotics in the management of kidney stone disease.

## Introduction

Oxalic acid and its oxalate salts appear in the blood and urine of animals and humans. Oxalate found in humans mainly originates from dietary sources containing oxalate, including strawberries, spinach, tea, and coffee (Duncan et al., 2002), while a small amount is formed by metabolizing glycine, ascorbic acid, and glyoxylate (Holmes and Assimos, 1998). Humans do not harbor enzymes to metabolize oxalate but micro-organisms in the gut can degrade oxalate or assimilate it into the urinary system. Small absorbance causes several pathological disorders, such as hyperoxaluria, renal failure, and kidney stones (Campieri et al., 2001). Kidney stones affect between 3% and 20% of people worldwide, and many patients relapse from the disease. Calcium oxalate, calcium phosphate, and uric acid are the major components that form kidney stones (Knight et al., 2007).

Composition and functionality of the human gut microbiota are correlated with kidney stones. The fecal microbiota of kidney stones formers showed reduced biodiversity and reduced expression of genes involved in oxalate degradation (Ticinesi et al., 2018). Oxalate-metabolizing gut bacteria have an important function in limiting oxalate absorption and reduce oxalate levels (Robijn et al., 2011). These bacteria are known as “oxalotrophic” bacteria, because they utilize oxalate as a sole carbon source. Generally, most of them are facultative hydrogen-oxidizing chemolithoautotrophs and/or facultative methylotrophs (Sahin, 2003). These bacteria can further be classified into “generalists” (ferment oxalate and other substrates) and “specialists” (use only oxalate as sole source) (Sahin, 2003). *Oxalobacter formigenes*, which belongs to the “specialists” group was first reported in humans with the function of oxalate degradation (Allison et al., 1985). The oxalate catabolism function has also been described in human gut bacteria from *Bifidobacterium*, *Enterococcus faecalis*, *Eubacterium lentum*, *Escherichia coli*, *Lactobacillus*, and *Providencia rettgeri* (Abratt and Reid, 2010). Two key enzymes, formyl-coenzyme A transferase (FCR) and oxalyl-coenzyme A decarboxylase (OXC), play a critical role in oxalate degradation. FCR catalyzes the reaction of transferring a CoA moiety to stimulate oxalic acid. Oxalyl-CoA is transformed into formyl-CoA and CO_2_ by OXC, which is a thiamine PPi-dependent decarboxylation reaction (Lewanika et al., 2007).

OXC (EC:4.1.1.8) belongs to the carboxy-lyases family. The structure of OXC comes from *E. coli* and *O. formigenes* (Berthold et al., 2005; Werther et al., 2010). The monomer of the enzyme consists of three domains of α/ β types, which are the pyrimidine (PYR) domain, the regulatory (R) domain, and the pyrophosphate (PP) domain (Werther et al., 2010). The enzyme forms a tetrameric structure and binds one thiamine pyrophosphate (TPP) and one metal ion (usually magnesium) per subunit as cofactors. The enzyme also binds ADP, which is essential for maximum decarboxylase activity through stabilizing the conformation of OXC (Berthold et al., 2007). OXC from *Bifidobacterium lactis* and *L. acidophilus* have also been studied (Federici et al., 2004; Azcarate-Peril et al., 2006). These enzymes share high sequence similarity (>45%) (Federici et al., 2004) suggesting that they share the same enzyme catalytic mechanism.

Analysis of the gut microbiota composition and the functionality of kidney stones in patients shows that there is a gut–kidney axis in the modulation of kidney stones formation, which is not limited to *O. formigenes*; however, OXC is still the pivotal enzyme for oxalate degradation (Ticinesi et al., 2018). Taking advantage of a multitude of bacterial genomes and metagenomes that have been sequenced, we analyzed presence, classification, and phylogenesis of OXC in various organisms in the current study. We further analyzed the abundance of the enzyme in the human microbiome, which is not limited to gut. Finally, two enzymes were purified and characterized. The results showed that OXCs are widely distributed in bacteria and human microbiomes. Bacteria with OXCs varied in ecological niches in human body even OXC are highly conserved during evolution.

## Materials and Methods

### OXC Mining and Sequence Analysis

OXC sequences were retrieved from the InterPro database^1^. The fragment sequences were removed and all the sequences were annotated by Pfam database^2^ to confirm the presence of OXC domain. Enzyme Function Initiative-Enzyme Similarity Tool (EFI-EST) was used to construct the protein sequence similarity networks (SSNs) (Gerlt et al., 2015), which were visualized by Cytoscape 3.3 (Shannon et al., 2003). Multiple alignments of OXC protein sequences were performed using the MAFFT v7 program (Katoh et al., 2017). MEGA7 was used to conduct phylogenetic trees based on MAFFT alignments using ML methods (bootstrapping iterations = 1000) (Kumar et al., 2016). The *dN* and *dS* substitution rates for OXC were evaluated using Java Codon Delimited Alignment (JCoDA) with a jump size of 1 codon and a sliding window of 25 codons (Steinway et al., 2010).

### Determination of OXC Abundance in Human Meta-Omics Data

The abundance of OXC in human metagenomes and metatranscriptomes was determined by Short, Better Representative Extract Dataset (ShortBRED) (Kaminski et al., 2015). Briefly, with the default parameters, ShortBRED-Identify was conducted to identify representative peptide markers for the protein OXC family. Once markers were obtained, ShortBRED-Quantify was occupied to map metagenomic reads against these markers to determine the relative abundance based on the reads per kilobase million (RPKM). Shotgun metagenomic reads for 380 metagenomes were retrieved from the HMP (Human Microbiome Project) website^3^.

### OXC Expression and Purification

The OXCmm gene of *Mycobacterium mageritense* (Uniprot ID: X5KV65) was synthesized by BGI (Shenzheng, China) technology, cloned into pET28a vectors, and transformed into *E. coli* BL21 (DE3) cells. The cells were cultured to OD_600_ = 0.7. Isopropyl β-D-1-thiogalactopyranoside (IPTG) was added and cultured continuously for 4 h. The harvested cells were lysed by ultrasound. The OXCmm protein was eluted by Ni-NTA resin column (American bridging root) with imidazole gradient (20–250 mm) until all resin-binding proteins were eluted. Dialysis was performed with 50 mM Tris HCl buffer (pH 8) containing 300 mm NaCl and 20 mm beta-mercaptoethanol. Bradford was used to estimate the protein concentration of bovine serum albumin (BSA) (Bradford, 1976).

### OXCmm Activity Assay

The activity of OXCmm was determined by capillary electrophoresis with the consumption of OX-CoA as the substrate. In 50 mM HEPES buffer (pH 6.8), thiamine pyrophosphate, 0.5 mM MgCl_2_, 0.2 mM oxaloacetate CoA, and 0.5 mM NAD^+^ were added. The purified OXCmm of 10 ng/uL was added and left to incubate for 10–60 min at 37°C. As mentioned above (Federici et al., 2004), the HPCE 3D system from Agilent Technologies (Waldbronn, Germany) was used to determine CO-CoA synthesis from coenzyme A (CoA) and thiocarbic acid by capillary electrophoresis (Quayle, 1962).

### Site-Directed Mutagenesis of Oxc

The primers were as follows: E62A, forward, 5′- GGC TTC CGG CAC GCC AGC GAC GCC GGT -3′; reverse, 5′- ACC GGC GTC GCT GGC GTG CCG GAA GCC -3′; Y126A, forward, 5′- CAA CGG GGT GAC GCC GAG GAG CTG GAT -3′; reverse, 5′- ATC CAG CTC CTC GGC GTC ACC CCG TTG -3′; E127A, forward, 5′- CGG GGT GAC TAC GCC GAG CTG GAT CAG-3′, reverse, 5′- CTG ATC CAG CTC GGC GTA GTC ACC CCG -3′; Y485A, forward, 5′- AAC GGT GGC GTC GCC CGG GGC GAC GGA -3′, and reverse, 5′- TCC GTC GCC CCG GGC GAC GCC ACC GTT -3′. The PET28-OXC plasmid was used as a DNA template for PCR amplification, and *Dpn*I was used to digest the PCR mixture and transform it into *E. coli*. All mutants were confirmed by BGI (Shenzhen, China) DNA sequencing. The method of protein purification was the same as that of wild-type.

## Results

### Classification and Distribution of OXCs

**FIGURE 1:**
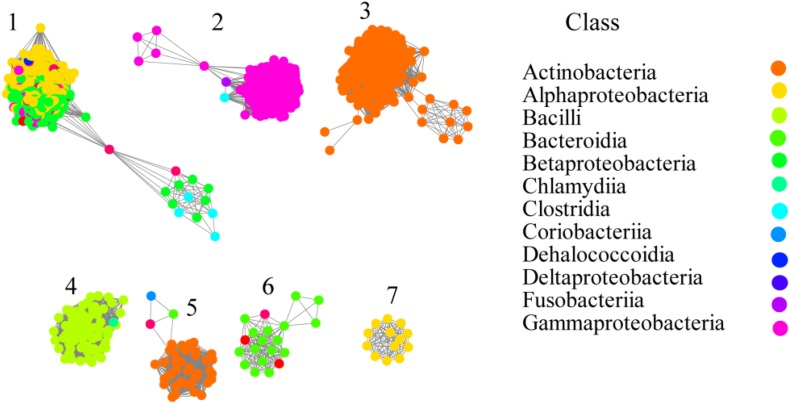
Networkanalysis of oxalyl-coenzyme A decarboxylase (OXC) based on sequence similarity. Each node represents one protein. The protein sequences are listed in the Supplementary Sheet. Edges are shown with BLASTP e-values below 10^–180^. A cluster was sequentially labeled. Nodes from the same taxonomic groups (Class level) in the network have been painted the same color. The colors corresponding to each class are listed at the right section. The experimentally characterized OXC from *Oxalobacter formigenes* (Uniprot ID: P40149; Cluster 1), *Escherichia coli* (Uniprot ID: P0AFI0 or P0AFI1; Cluster 2), *Lactobacillus acidophilus* (Uniprot ID: A0A378H7I7; Cluster 4) *Bifidobacterium animalis* (Uniprot ID: B8DWU2; Cluster 5) are enlarged and circled.

From the relationship between availability and phylogeny, the gene distribution of prokaryotes can be analyzed comprehensively. In order to understand OXC in depth, currently available genomes of different species were screened in the UniProt and InterPro database for the presence of OXC. In total, 1396 protein sequences were collected (Supplementary Sheet) and OXC homologs were found to be in many bacteria but not in archaea or other eukaryotes. These proteins can be found in the following classes: Actinobacteria, Alphaproteobacteria, Bacilli, Bacteroidia, Betaproteobacteria, Chlamydia, Clostridia, Coriobacteriia, Dehalococcoidia, Deltaproteobacteria, Fusobacteria, and Gammaproteobacteria. Among them, OXC was mainly distributed in Actinobacteria (23%), Alphaproteobacteria (23%), Gammaproteobacteria (26%), and Betaproteobacteria (18%). These proteins showed high sequence similarity because they form a closed network if the SSNs were constructed using a protein sequence identity cutoff of 50% (Supplementary Figure S1A). When the cutoff of the protein sequence identity was set to 60%, four clusters were formed as some proteins from Actinobacteria, Bacilli, and Betaproteobacteria can be separated (Supplementary Figure S1B). When the cutoff was further increased to 70%, all of the OCX homologs separated to seven clusters (Figure 1). In this study, we employed the criteria to classify OXCs in further research because the classification is in accordance with the taxonomic distribution. For example, the proteins in Cluster 2 mostly occurred in Gammaproteobacteria; the OXC from *E.coli* was in Cluster 2 and the proteins in Actinobacteria were mainly found in Clusters 3 and 5; the OXC from *Bifidobacterium lactis* was in Cluster 5. The proteins in Cluster 4 mostly occurred in the *Bacilli* class, including the protein from *L. acidophilus*. Few proteins were in Clusters 6 and 7, which were from Betaproteobacteria and Alphaproteobacteria, respectively. In contrast, the proteins in Cluster 1 were from diverse bacteria classes, including Alphaproteobacteria, Betaproteobacteria, and Gammaproteobacteria. OXC From *O. formigenes* was in Cluster 1.

### Gene Context of OXCs With FRC

**FIGURE 2:**
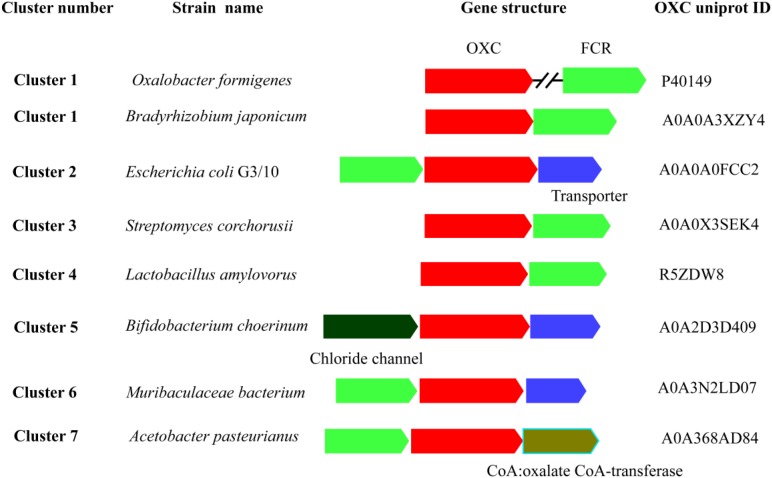
Schematic view of the genomic context of oxalyl-coenzyme A decarboxylase (OXC). The bacteria of the OXCs are listed on the left. The UniProt IDs of the OXCs are listed on the right. The S OXCs or FCR are shown in red and blue color, respectively. The annotated names of other genes are shown by different color and shown under the genes.

OXC and FRC worked together to metabolize oxalic acid. To better understand the degradation of oxalic acid by the two enzymes, a gene context analysis was carried out to elucidate their relationship. The OXCs from Cluster 1 were from a variety of bacteria, the gene context of OXCs in the cluster was also diverse (Figure 2). In *O. formigenes*, the OXC does not form an operon with FRC, however, the two genes cluster together in *Bradyrhizobium japonicum*, which is also located in Cluster 1. The gene context formed by the two genes was also detected in Cluster 3, Cluster 4, and Cluster 7. In Cluster 2, OXC, FRC together with a transporter gene form an operon, suggesting the transporter plays an important role in oxalic acid transport. A similar gene context can be observed in Cluster 5, which is linked with a chloride chancel, suggesting there are diverse ways to transport oxalic acid. These analyses suggested that the gene context of different clusters showed quite similar and the two key enzymes for oxalic acid are generally exit together.

### Phylogenetic Analysis and Evolutionary Rate of OXCs

**FIGURE 3:**
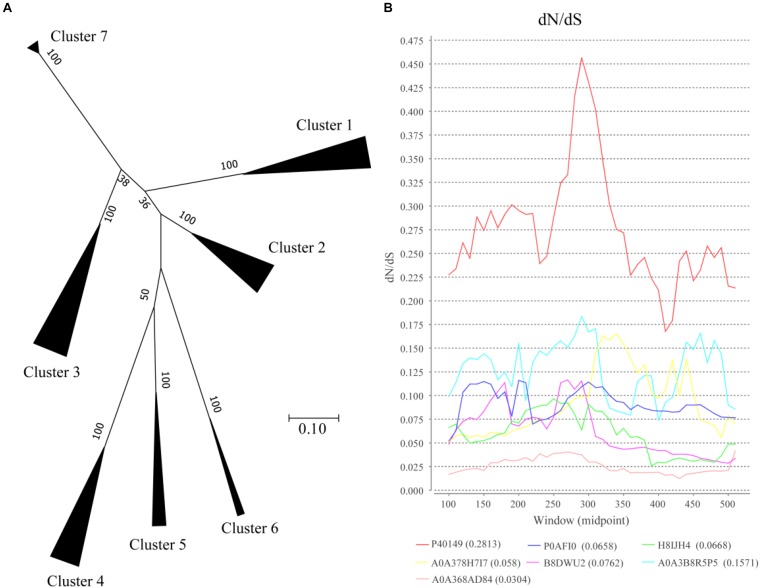
Evolutionary analysis of oxalyl-coenzyme A decarboxylase (OXC). **(A)** Phylogenetic analysis of the oxalyl-coenzyme A decarboxylase (OXC) family. Unrooted maximum likelihood phylogenetic tree of OXC proteins. All taxa from each group in Figure 1 were clipped from the tree and replaced by a triangle, whose width is proportional to the number of taxa in that group. The numbers at the branches are confidence values based on bootstrap method calculated using 1000 bootstrap replications. The scale bar in the figure represents 0.2 substitutions per site. **(B)** *dN/dS* analysis of OXC of 7 enzymes from the 7 clusters as shown by sliding-window. The *dN/dS* rate of different clusters are shown at the bottom of the figure. The Uniprot ID of these enzymes were listed in the end of the figure. The corresponding strains of the enzymes are: P40149: *Oxalobacter formigenes* (Cluster 1), P0AFI0: *Escherichia coli*(Cluster 2), H8IJH4: *Mycobacterium intracellulare* (Cluster 3), A0A378H7I7: *Lactobacillus acidophilus* (Cluster 4), B8DWU2: *Bifidobacterium animalis* (Cluster 5), A0A3B8R5P5: *Porphyromonadaceae bacterium* (Cluster 6), A0A368AD84: *Acetobacter sp.* BCRC 14118 (Cluster 7).

To analyze the evolutionary relationships among clusters, protein sequences in each cluster based on SSNs assignment were used for further analysis (Figure 3A). In the phylogenetic tree, Clusters 4–6 were closed even though the proteins in the clusters were from different bacteria classes, including Bacilli, Actinobacteria, and Betaproteobacteria. Both the proteins from Clusters 3 and 5 were from Actinobacteria, but they are relatively far in the phylogenetic tree suggesting that the proteins in the two clusters were not close during evolution. On the other hand, the proteins in Cluster 1 were from three bacteria classes of bacteria but they formed a closed branch suggesting these proteins share a common ancestor and gene transfer occurred during evolution. These results suggest that OXC genes are conserved more during evolution and gene transfer occurred during evolution, which is not a common event.

The ratio of non-synonymous-to-synonymous divergence (*dN*/*dS* = ω) of OXC genes were further examined to measure the selection pressure of the enzymes (Figure 3B). One representative sequence from each cluster were selected and the *dN/dS* ratio was measured. The results indicated that the *dN/dS* ratios for 7 OXC genes from the 7 clusters are <1, indicating that the OXC are evolved under negative selection. On the other hand, the *dN/dS* ratios of the 7 genes are different, especially the gene from Cluster 1, which shows the highest *dN/dS* ratio. However, the ω values for the region located between 250 amino acid to 300 amino acids were relatively higher than the value of other regions in all the clusters. The *dN/dS* ratios from other enzymes in the same cluster showed similar pattern (data not shown). These findings suggested that OXCs in different clusters share a common evolutionary pattern even they are under different selection pressure.

### Abundance of OXCs in the Human Microbiome

**FIGURE 4:**
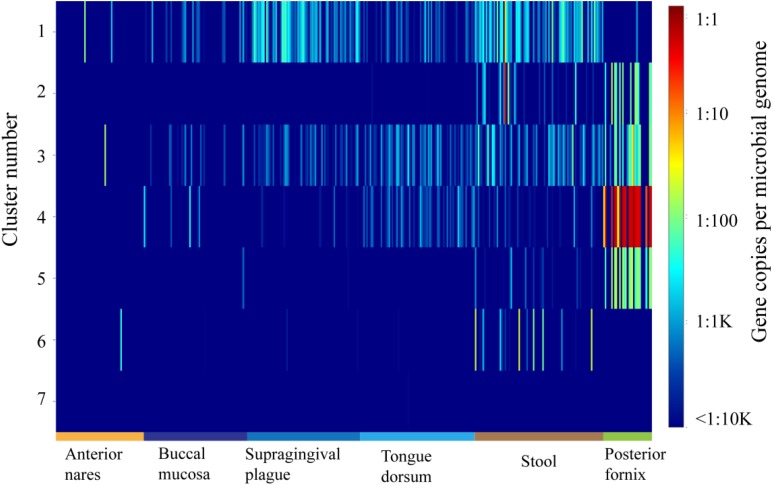
The abundance and distribution of the 7 oxalyl-coenzyme A decarboxylase (OXC) clusters in 378 HMP metagenomes from six body sites as quantified using ShortBRED.

ShortBRED was further used to demonstrate the abundance of each group in the OXC family in metagenomes during the HMP (Kaminski et al., 2015). The abundance of OXC was found across multiple body sites, from aerobic (skin and vaginal) to microaerobic (oral) and to anaerobic (gut) environments (Figure 4). On the basis of ShortBRED, all of the OXCs groups can be found in the HMP except Cluster 7. Among the seven clusters, Cluster 1 is the most abundant member of the human guts and the enzymes in Cluster 1 also highly occurred in oral sites, including the sites of the buccal mucosa, supragingival plaque, and tongue dorsum. However, the abundance of Cluster 1 was relatively low in vaginal sites. In contrast, the enzymes in Clusters 2–5 were relatively low in the guts but high in vaginal sites. Cluster 6 was low in both the guts and vaginal sites. This data suggested that the bacteria with OXCs from different clusters prefer different ecological niches in human body.

### Activity Assay of OXCmm

**FIGURE 5:**
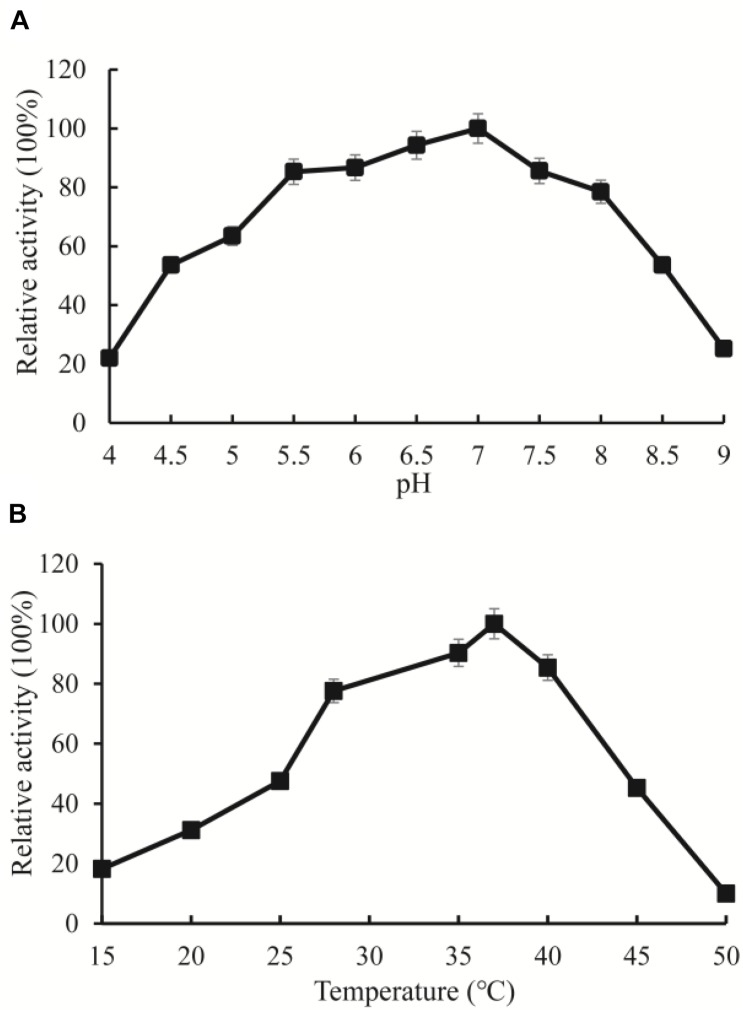
Enzyme activity assays of OXC from *M. mageritense* (OXCmm). **(A)** Optimal pH of OXCmm activity. Different buffers were used for the different pH solutions in this assay. Sodium acetate buffer was used for pH 4.0–4.5; Phosphate buffer was used for pH 5.0 and 7.5; Tris buffer was used for pH 8.0. **(B)** Optimal temperature of OXCmm activity.

Among the high abundance of OXC clusters, the enzymes in Cluster 3 have not been experimentally characterized. To investigate the function of the enzymes in this cluster, we synthesized OXCmm in the cluster. Theoretically, *OXCmm* ORF codes for the protein of 576 aa, with a pI of 5.51, and molecular weight (MW) of 59.76. The optimal pH and temperature of the recombinant OXCmm were measured and displayed in Figure 5. To study the pH effect on OXCmm, the decarboxylase activity was measured from pH values of 4.0 to 9.0 at 37°C. OXCmm showed high activity between the pH values of 6.0–8.0. OXCmm activity increased with the induction of pH and reached the highest at pH 7.0, with half activity at pH 8.0. Temperature ranging from 10–60°C also had an effect on OXCmm activity at pH 7.0. Above 15°C, the activity of the recombinant enzyme increased with temperature elevation. The optimum temperature for the enzyme was around 37°C (Figure 5).

### Enzymatic Activity Effected by Point Mutation

**FIGURE 6:**
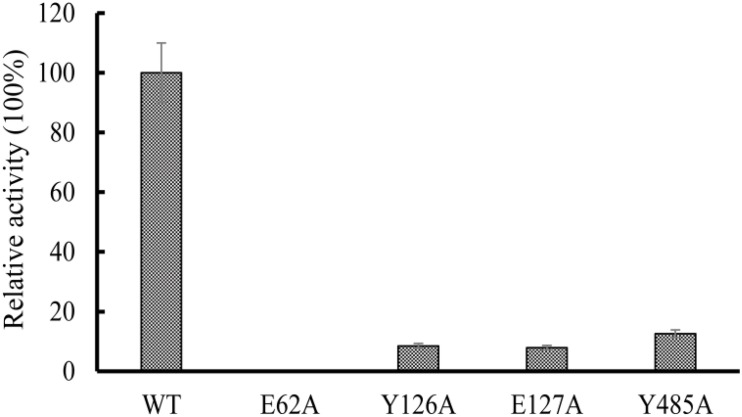
Relative activity of wild-type OXC from *M. mageritense* (OXCmm), E62A, Y126A, E127A, and Y485A. All assays were repeated three times. The error bars indicate the standard deviation of the independent measurements.

Multiple sequence alignments showed that the amino acids sequence of OXCmm shows 54% identity to the sequence of OXC from *O. formigenes* (Supplementary Figure S2). The results further revealed E62 is highly conserved, which is responsible for proton transfer (Berthold et al., 2007). To confirm the function of E62, the amino acid was mutated to Ala. Y126, E127, and Y485 had the function to position the oxalyl group of the substrate at the active site. These amino acids were also mutated to Ala. The mutant enzymes (E62A, Y126A, E127A, and Y485A) were expressed and purified. The activities of these mutant and wild-type enzymes were determined (Figure 6). The results showed that no decarboxylase activity of E62A could be detected and Y126A, E127A, and Y485A have about 10% activity of the wild-type OXCmm. The mutant activity assay was similar to the activity of the mutants in OXC from *O. formigenes* suggesting that the two enzymes share a common mechanism even though they are from different clusters.

## Discussion

High levels of oxalate in humans can have a detrimental, corrosive effect. It can cause a range of medical pathologies including hyperoxaluria and renal failure (Hoppe et al., 2009), calcium oxalate urolithiasis (Campieri et al., 2001), and cardiomyopathy (Van Driessche et al., 2007). Hyperoxaluria is characterized by extremely high levels of urinary oxalate, which can lead to urolithiasis (stone formation). Recent studies showed that there is the existence of a gut–kidney axis in nephrolithiasis physiopathology (Stern et al., 2016; Ticinesi et al., 2018). oxalate metabolizing bacterial species (OMBS) harboring OXC genes in the human gut have been considered to play important roles in kidney stone formation. Our study showed that OXCs were widely distributed in the gut and other environments. These finding can extend the knowledge about OXCs from their diversity, evolution, and to biological functions.

Oxalate oxidase, decarboxylase, FCR, and OXC are the key oxalate-degrading enzymes. Oxalate decarboxylases and oxalate oxidases are members of the cupin superfamily of proteins and the two enzymes show high similarity at the amino acid level (Burrell et al., 2007). The genes encoding the four enzymes were widely present in the healthy gut microbiome on the basis of the analysis of 660 subjects (Liu et al., 2020). However, the genes encoding FCR and OXC were substantially more prevalent and more abundant than the genes encoding oxalate oxidase and decarboxylase in the gut. Among the 660 subjects, OXC can be detected in the metagenome of 554 subjects (84%) and FCR can be detected in 581 subjects (88%) (Liu et al., 2020). These data suggested that OMBS with OXC and FCR are the predominant bacteria in the healthy human gut. Our study further classified these OXCs into 7 clusters and showed that Cluster 1 is the most abundant member of the human guts, which gave further precise information that bacteria with the OXCs from Cluster 1 are the dominant OMBS in the gut of healthy humans.

Gut microbiota plays important roles in the physiopathologic gut–kidney axis. A multi-species bacterial network could keep healthy oxalate homeostasis to inhibit urinary stone disease (Miller et al., 2019). Microbial dysbiosis of eubacterial, archaeal and eukaryotic components occurred in the patients with recurrent oxalate kidney stones (Suryavanshi et al., 2018). Several studies showed that the abundance of Firmicutes increased while the abundance of Bacteroidetes decreased in the gut of humans (Stern et al., 2016; Suryavanshi et al., 2018; Tang et al., 2018). In addition, both oxalate metabolizing enzymes and oxalate metabolizing bacterial species were found augmented in the gut of patients with hyperoxaluria, however, along with well-studied bacterium, *Oxalobacter formigenes*, other genera had significant difference in the symptomatic hyperoxaluria conditions (Suryavanshi et al., 2016), suggesting that there may be other commensal bacteria possessing the ability to utilize oxalate. Our study showed that OXC, the main enzyme for oxalate degrading, was widely distributed in bacteria and in total, 1397 enzymes were found. The bacteria with these enzymes may be the potential candidates and further experiments can be performed to prove the hypothesis.

OXC active bacteria in the human gut have been considered as probiotics in the management of kidney stone disease; however, most studies have demonstrated that probiotic products containing *O. formigenes* and lactobacilli with oxalate-degrading activity were not able to influence oxalate excretion and the nephrolithiasis course. Furthermore, *in vivo* oxalate degradation activity may be lower for *O. formigenes* (Duffey et al., 2011; Siener et al., 2013a, b). The oxalate-degrading activity may in fact be shared by a large number of taxa, influencing each other in a complex metabolic network, and not rely solely on single player or a limited number of species. Thus, introducing other alternative OMBS in the gut environment by administering a probiotic may a promising way to produce a significant overall endoluminal oxalate degradation. Our study showed that the OXC active bacteria in different clusters may occupy diverse ecological niches. For example, the OXCs in Cluster 1 were relatively high in the gut, suggesting the bacteria harboring these OXCs could colonize well in the gut. On the other hand, the bacteria harboring these OXCs from Cluster 4 may colonize well in the vagina. These bacteria may serve as potential probiotics for improving health because they could colonize well in the corresponding organs.

The structure of OXCs from *O. formigenes* and *E. coli* has been resolved (Berthold et al., 2005; Werther et al., 2010). The two enzymes from different clusters showed similar folding. In the structure, the glutamic acid at N-terminus participates in proton transfer to promote the formation of the 1′,4′-iminopyrimidine tautomer of the TPP. Our mutagenesis analysis using OXCmm showed that mutation of the glutamic acid to alanine (E62A) absolutely abolished the enzyme activity, further confirmed the function of the amino acid in catalysis. In the structure of OXCs from *O. formigenes*, Tyr-120 and Glu-121 form hydrogen bonds with TPP to position the oxalyl group (Berthold et al., 2007). Mutation of the corresponding amino acid in OXCmm (Y126A and E127A) dramatically decreased enzyme activity. Tyr-483 in O. *formigenes* form a hydrogen bond to a water molecule to position the oxalyl group in the active site (Berthold et al., 2007). Our point mutation analysis also indicated that the activity of Y485A of OXCmm was decreased. OXCmm, OXCs from *O. formigenes* and *E. coli* are from different clusters, but these enzymes occupy the same conserved active sites, suggesting that OXCs are highly conserved during evolution.

OXC employs TPP as a cofactor. OXC is high related to acetolactate synthase in evolution, another TPP-dependent enzyme responsible for the biosynthesis of branched chain amino acids (Svedružić et al., 2005). We retrieved 1396 protein sequences to analyze the evolutionary relationship using SSN, phylogenetic tree and evolutionary rate. These analyses indicated that OXCs are relatively high conserved during under negative selection. Negative selection acts strongly against large differences and is important for proteins to preserve structure and function, suggesting OXC should play a vital role in bacterial metabolism. Gene transfer during OXC evolution occurred, however, the process happened in Cluster 1, but not other clusters. This analysis further suggested OXC is highly conserved during evolution.

## Conclusion

In conclusion, we retrieved 1396 OXC homologs and classified them into 7 clusters based on SSN. These proteins are conserved and suffered a positive selection evolution. The protein sequences in each cluster showed a quite different abundance in human body sites. One enzyme (OXC) from Cluster 3 was purified and mutated to study the catalytic mechanism, which showed that it occupies the same active sites with the enzymes from Cluster 1 and Cluster 3. These data suggested that OXCs are highly conserved during evolution and may provide an alternative avenue to use bacteria with OXC as probiotics to benefit patients suffering from kidney stone disease. However, it should be noted that there are several limitations in the study and further research can be performed: (1) Genome based phylogenetic analysis can be performed to reveal the single nucleotide polymorphism in *oxc* genes; (2) The activity of OXC in other clusters should be analyzed and compared; (3) The abundance of OXC in disease conditions should be further analyzed to reveal the contribution of OXC to human health.

## Data Availability Statement

The datasets analyzed in this study can be found in the InterPro database: http://www.ebi.ac.uk/interpro/entry/InterPro/IPR017660/.

## Author Contributions

TJ did the project design, data analysis, and wrote the manuscript. WC, LC, and YH did the evolutionary analysis. HZ and HM did the project design and wrote the manuscript.

## Conflict of Interest

The authors declare that the research was conducted in the absence of any commercial or financial relationships that could be construed as a potential conflict of interest.

## Footnotes

1 http://www.ebi.ac.uk/interpro/entry/IPR017660, 2019_02

2 https://pfam.xfam.org/

3 www.hmpdacc.org
