# Supplementary material for: Abundance, Functional, and Evolutionary Analysis of Oxalyl-Coenzyme A Decarboxylase in Human Microbiota
Source: Front Microbiol. 2020 Apr 23;11:672. doi: 10.3389/fmicb.2020.00672 (PMC7190790; doi:10.3389/fmicb.2020.00672)
Supplement: Supplementary file 1 [file Data_Sheet_1.PDF]

Supplementary Fig. 1

A

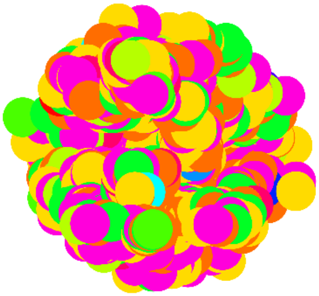

B

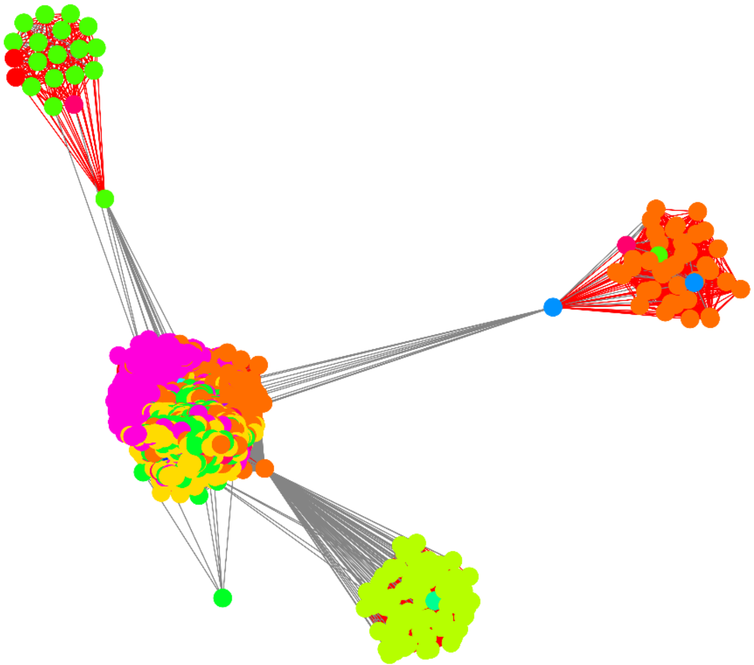

Supplementary Fig. 1. Network analysis of oxalyl-coenzyme A decarboxylase (OXC) based on sequence similarity. Each node represents one protein. The protein sequences are listed in the Supplementary sheet. Edges are shown if protein sequence identity was below below 50% (A) or 60% (B).

Supplementary Fig. 2.

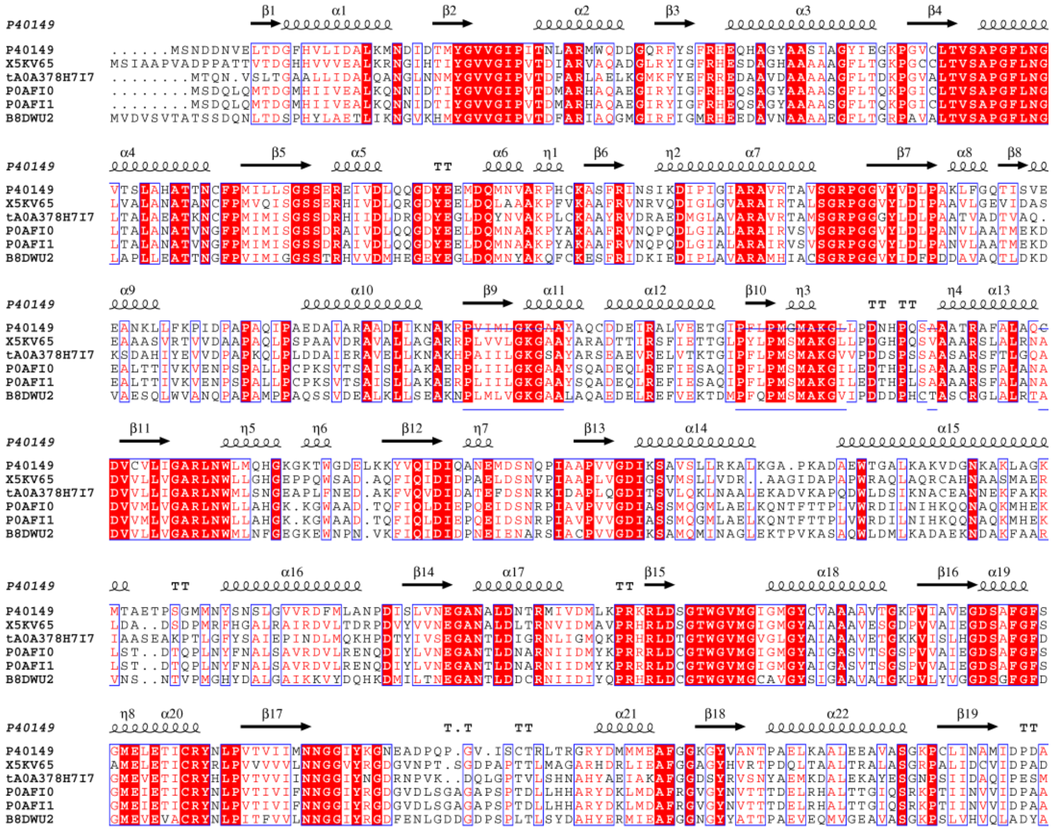

Supplementary Fig. 2. Multiple sequence alignment of oxalyl-coenzyme A decarboxylase (OXC) from *Oxalobacter formigenes* (P40149), *Mycolicibacterium mageritense* (X5KV65), *Lactobacillus acidophilus* (A0A378H7I7), *Escherichia coli* (P0AFI0), *Escherichia coli* (P0AFI1), and *Bifidobacterium animalis* (B8DWU2).
